# Adding pieces to the puzzle of differentiated-to-anaplastic thyroid cancer evolution: the oncogene E2F7

**DOI:** 10.1038/s41419-023-05603-8

**Published:** 2023-02-10

**Authors:** Mila Gugnoni, Eugenia Lorenzini, Italo Faria do Valle, Daniel Remondini, Gastone Castellani, Federica Torricelli, Elisabetta Sauta, Benedetta Donati, Moira Ragazzi, Francesco Ghini, Simonetta Piana, Alessia Ciarrocchi, Gloria Manzotti

**Affiliations:** 1 Laboratory of Translational Research, Azienda USL - IRCCS di Reggio Emilia, Reggio Emilia, Italy; 2 Department of Physics and Astronomy, University of Bologna, Bologna, Italy; 3 Department of Electrical, Computer and Biomedical Engineering, University of Pavia, Pavia, Italy; 4 Pathology Unit, Department of Oncology and Advanced Technologies, Azienda USL - IRCCS di Reggio Emilia, Reggio Emilia, Italy

**Keywords:** Oncogenes, Cancer genomics, Thyroid cancer

## Abstract

Anaplastic Thyroid Cancer (ATC) is the most aggressive and de-differentiated subtype of thyroid cancer. Many studies hypothesized that ATC derives from Differentiated Thyroid Carcinoma (DTC) through a de-differentiation process triggered by specific molecular events still largely unknown. E2F7 is an atypical member of the E2F family. Known as cell cycle inhibitor and keeper of genomic stability, in specific contexts its function is oncogenic, guiding cancer progression. We performed a meta-analysis on 279 gene expression profiles, from 8 Gene Expression Omnibus patient samples datasets, to explore the causal relationship between DTC and ATC. We defined 3 specific gene signatures describing the evolution from normal thyroid tissue to DTC and ATC and validated them in a cohort of human surgically resected ATCs collected in our Institution. We identified E2F7 as a key player in the DTC-ATC transition and showed in vitro that its down-regulation reduced ATC cells’ aggressiveness features. RNA-seq and ChIP-seq profiling allowed the identification of the E2F7 specific gene program, which is mainly related to cell cycle progression and DNA repair ability. Overall, this study identified a signature describing DTC de-differentiation toward ATC subtype and unveiled an E2F7-dependent transcriptional program supporting this process.

## Introduction

Anaplastic thyroid cancer (ATC) is a rare but highly aggressive form of thyroid cancer [1, 2]. By contrast, differentiated thyroid cancer (DTC) only rarely behave aggressively and develop distant metastasis. The genomic landscape of DTCs [3] and ATCs [4] have been described. While DTCs are characterized by high genomic stability and very low mutation density, ATCs are defined by a significant degree of genomic abnormalities that likely underscores their high mortality rate [5]. Still, in a percentage ranging from 8% to 80% of cases, in the same ATC, differentiated and de-differentiated components coexist, raising the hypothesis that specific molecular events trigger de-differentiation processes leading to ATC evolution from pre-existing DTCs [2, 6, 7]. This theory is sustained by several studies [8–10], but the molecular basis of this transition remains to be fully elucidated. Understanding the molecular pathogenesis of ATCs and the mechanisms leading to their aggressiveness is crucial for the development of more effective treatment strategies. A major limitation is represented by the poor availability of ATC samples for the analysis, determined by the rarity of these lesions and the advanced stage at diagnosis, which lessen the number of patients undergoing surgical resection. Despite some attempts to characterize ATC-specific signatures using published datasets to maximize sample number and statistical significance [11], the lack of a specific focus on DTC/ATC evolution, and the paucity of sample number still limit their value and need to be supported by broader analyses.

E2F proteins are a family of transcription factors (TFs), involved in the Retinoblastoma (Rb)-dependent pathway that control cell cycle progression by orchestrating a precise pattern of gene expression [12]. E2F7 and E2F8 are atypical members of this family and function in a Rb-independent manner [13, 14]. Known for its activity as transcriptional repressor, E2F7 has been linked to cell cycle inhibition competing with E2F1 for the same target genes [15–18]. E2F7 also partakes to stress response, DNA damage and cell survival regulation, even if its role in these processes is only partially clarified [19, 20]. Confined to the definition of E2F1 antagonist, the role of E2F7 in cell cycle regulation has been limited for long time to its canonical function as transcriptional repressor and onco-suppressor in some cancer settings.

In this study, we constructed a model to explain ATCs origin integrating gene expression microarray data from different studies. We confirmed, using Nanostring technology, that ATC represents a form of progression of DTC, and we identified a specific gene module as the building block of this evolution. We found E2F7 as master regulator coordinating this non-canonical, context specific, pro-oncogenic gene expression program fundamental for ATC aggressiveness and progression, defining a crucial step in DTC to ATC evolution.

## Results

### An E2F7-centered, cell cycle related gene module defines DTC to ATC evolution

**Fig. 1:**
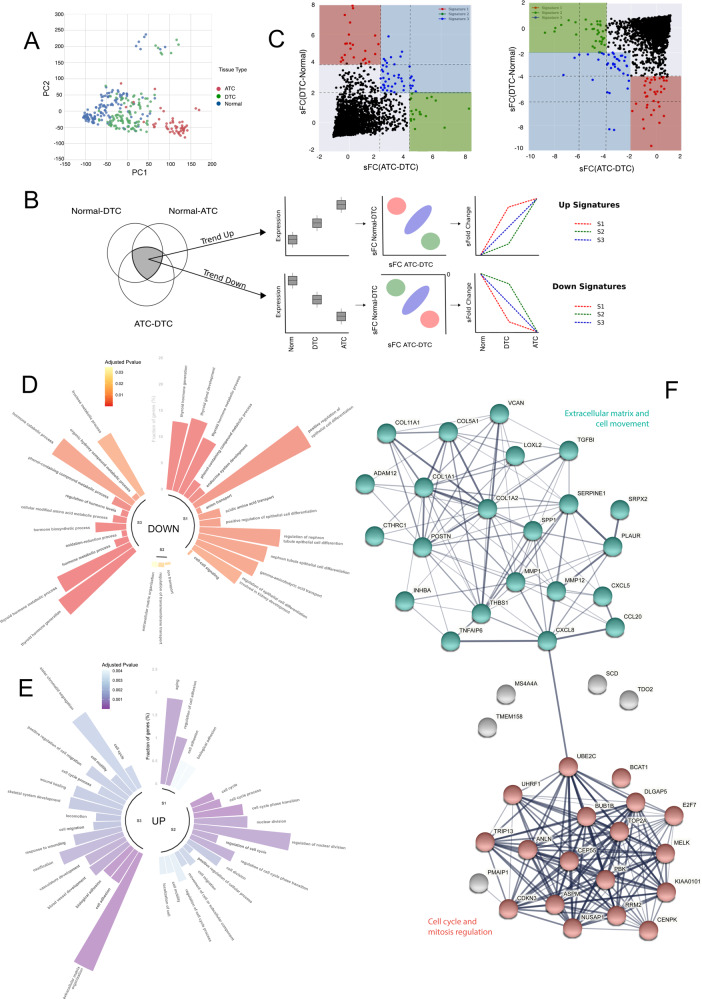
A cell-cycle related gene signature describes the DTC-ATC transition. **A** Principal component analysis applied to the gene expression dataset composed by normal, DTC and ATC thyroid tissues. **B** Diagram showing the steps in the definition of the gene signatures. The Trend Up and Trend Down gene lists were subsequently divided into signatures S1, S2 and S3, according to their standardized fold change values in the normal-DTC and ATC-DTC comparisons. **C** Scatter plot depicting the selection of three signatures from the Trend Up and Trend Down gene lists. The axes show standardized fold change values in the comparisons DTC-Norm (Y axis) and ATC-DTC (X axis). The shaded areas highlight the cutoffs used in the definition of each signature. **D**, **E** GO enrichment analysis for genes differentially expressed in the ATC vs DTC comparisons. Figures show the main altered pathways for each of the three gene signatures identified, divided as Trend Down **D** and Trend Up **E**. Color scale indicates p-value, the fraction of genes enriched in each pathway is plotted. **F** STRING protein–protein interaction prediction among the genes of signatures Trend Up S2 and S3. In coral the genes enriched in GO terms related to cell cycle and mitosis regulation, while in turquoise the genes enriched GO terms related to extracellular matrix and cell movement (see also Supplementary Table S7).

Owning to their rarity, the molecular mechanisms underlining ATC evolution in far from being elucidated. In a percentage of cases from 8% to 80% (depending on series), ATC coexists with a DTC component within the same tumor, raising the hypothesis that ATCs originate from the progressive de-differentiation of pre-existing DTCs [7]. We explored the causal relationship between DTC and ATC using gene expression data. To overcome the limitation of poor ATC samples availability, we performed a meta-analysis combining available profiles from different studies. We analyzed 279 gene expression profiles, from 8 GEO patient samples datasets, including normal thyroid (*n* = 127), DTC (mainly papillary thyroid cancer, PTCs) (*n* = 102) and ATC (*n* = 50) samples (Supplementary Table S1). After data normalization and standardization, we clustered samples according to the expression levels of the 150 probes with highest variance across the entire dataset. We observed that patients did not group according to the study of origin, supporting the absence of strong batch effects and showing that the assembled dataset was suitable for evaluating the differences and similarities among tissue types (Supplementary Fig. S1A). Principal component analysis (PCA) on the entire dataset demonstrated that samples divided into three main clusters that largely corresponded to normal, DTC and ATC samples (Fig. 1A, Supplementary Fig. S1B). DTCs showed the widest distribution spreading in between normal and ATC samples. Only 14 samples showed anomalous segregation at the top of the PCA. These samples belong to the same dataset (GSE3678) (Supplementary Fig. S1C) and were excluded from downstream analyses.

To follow gene expression changes across a normal-DTC-ATC linear evolution, differentially expressed genes (DEGs) were divided in Trend Up (2544 genes) and Trend Down (3085 genes). These lists were further subdivided based on their absolute standardized fold change (sFC) values (Fig. 1B, C). Genes with high sFC in the DTC-normal comparison and low sFC in ATC-DTC comparison were defined as Signature 1 (S1), those with the inverse pattern were defined as Signature 2 (S2), and those equally high deregulated in both comparisons were defined as Signature 3 (S3) (Supplementary Table S6). In the Trend Up, 25 genes resulted in the S1, 17 in the S2 and 29 genes in the S3. In Trend Down, 22 genes resulted in the S1, 29 in the S2 and 31 in the S3. Gene ontology (GO) analysis performed on Trend Down genes, both for S1, S2, and S3 list, were enriched in thyroid functionality and hormone metabolism. S1 genes highlighted also alteration of the epithelial cell differentiation processes (Fig. 1D). On the other hand, Trend Up genes were strongly related to cell migration, extracellular matrix organization and cell cycle. Also of note the association of S3 genes with ossification and skeletal development since thyroid cancer aggressiveness and mortality are reportedly correlated with the reactivation of embryogenetic pathways involved in skeletal development which contribute to the setting of distant bone metastases [21, 22] (Fig. 1E). Since thyroid functions are already reported to be de-regulated in thyroid carcinomas, we focused on Trend Up genes, and in particular on S2 and S3 signatures which suggest a specific landscape underlying the DTC to ATC evolution. Disease-associated genes generally show propensity to aggregate into the same interactome neighborhood defining disease-associated gene modules [23]. Thus, we mapped the 46 genes of the Trend Up S2 and S3 signature in the STRING protein–protein interactions database. We observed that 40/45 (88.9%) genes aggregate into a sub-network defining an ATC-associated gene module (Fig. 1F). Within this module, two main clusters of relationship were identified. One cluster (turquoise) was enriched for genes controlling extracellular matrix and cell movement. The largest cluster (coral) was enriched in genes related to cell cycle and mitosis regulation (Supplementary Table S7). Most Trend Up S3 genes were located in the matrix-interaction and cell microenvironment cluster. By contrast, the S2 genes largely located within the cell cycle related cluster, even if this differential distribution was not statistically significant (Fisher exact-test p-value > 0.05).

These data indicate that phenotypical differences between DTC and ATC are marked by the gained expression of genes involved in cell cycle progression, proliferation, and migration, in line with ATC higher aggressiveness and rapid progression. Interestingly, E2F7 was the unique transcription factor among the Trend Up genes. This is in contrast with its reported role as tumor suppressor and suggest a possible atypical function in this context.

### Validation of the trend up signature in ATC patients’ samples

**Fig. 2:**
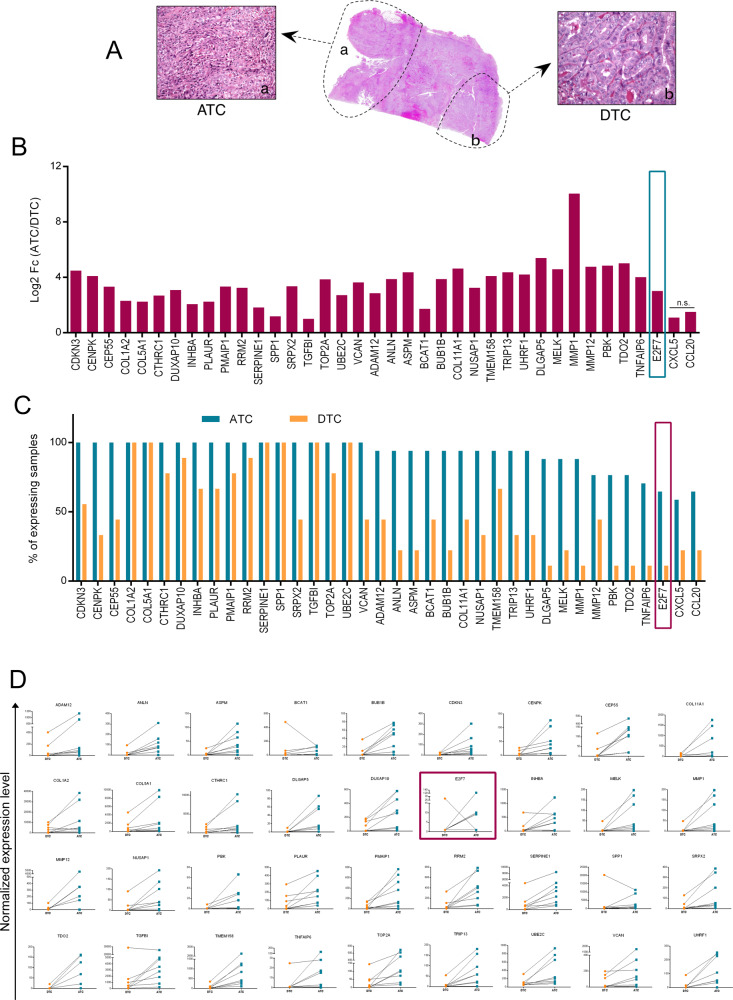
In vivo validation of Trend UP S2 and S3 signatures in ATC patients. **A** Representative example of components separation in ATC mixed samples used for the analysis. **B** Gene expression analysis of Trend Up S2 and S3 genes in ATC samples. The bars represent the Fold Change (log2) of the difference between gene expression in ATC and in DTC. All the values are statistically significant (adjusted *p*-value < 0.05) except where “n.s.” is reported. **C** Percentage of samples (ATC and DTC) expressing the genes validated through Nanostring. **D** Normalized expression levels of indicated genes in matched ATC and DTC from the same patient. The expression of each gene in the indicated tissues is normalized on 4 housekeeping genes.

We validated the Trend Up S2 and part of the S3 genes in a validation cohort of 23 human surgically resected ATCs collected in our Institution from 1975 to 2015, using the nCounter Nanostring technology. Considering the rarity of this type of tumor and the limited surgical procedures performed on these patients, this cohort represents one of the largest ever analyzed. Clinical information of these samples is available in Supplementary Table S8. For 14 of these samples, the DTC component was also available for the analysis. In these mixed tumors, ATC and DTC components were micro-dissected and analyzed as separate samples (Fig. 2A). Gene expression profile was obtained for 18 patients excluding samples with poor RNA quality (Supplementary Table S2, S9). Eight patients having both DTC and ATC components and 10 patients for whom only one of the two components was evaluable. 80% (*N* = 38) of the ATC-associated genes, including E2F7, were validated as significantly up-regulated in ATCs as compared with DTCs (Fig. 2B and Supplementary Table S9). We evaluated the expression of each gene of the validated signature in ATC and DTC specimens and found that E2F7 is one of the genes with the highest difference between ATC and DTC samples (Fig. 2C). Finally, we compared the expression level of the validated genes in matched ATC and DTC samples, derived from the same lesions (*n* = 8). This further confirmed the consistent increased expression of these genes, including E2F7, in the anaplastic component compared to the differentiated counterpart (Fig. 2D).

These analyses highlighted the existence of an essential core of genes whose upregulation marks the transition from DTC to ATC within the same lesion.

### E2F7 is essential for proliferation and migration of ATC cells

**Fig. 3:**
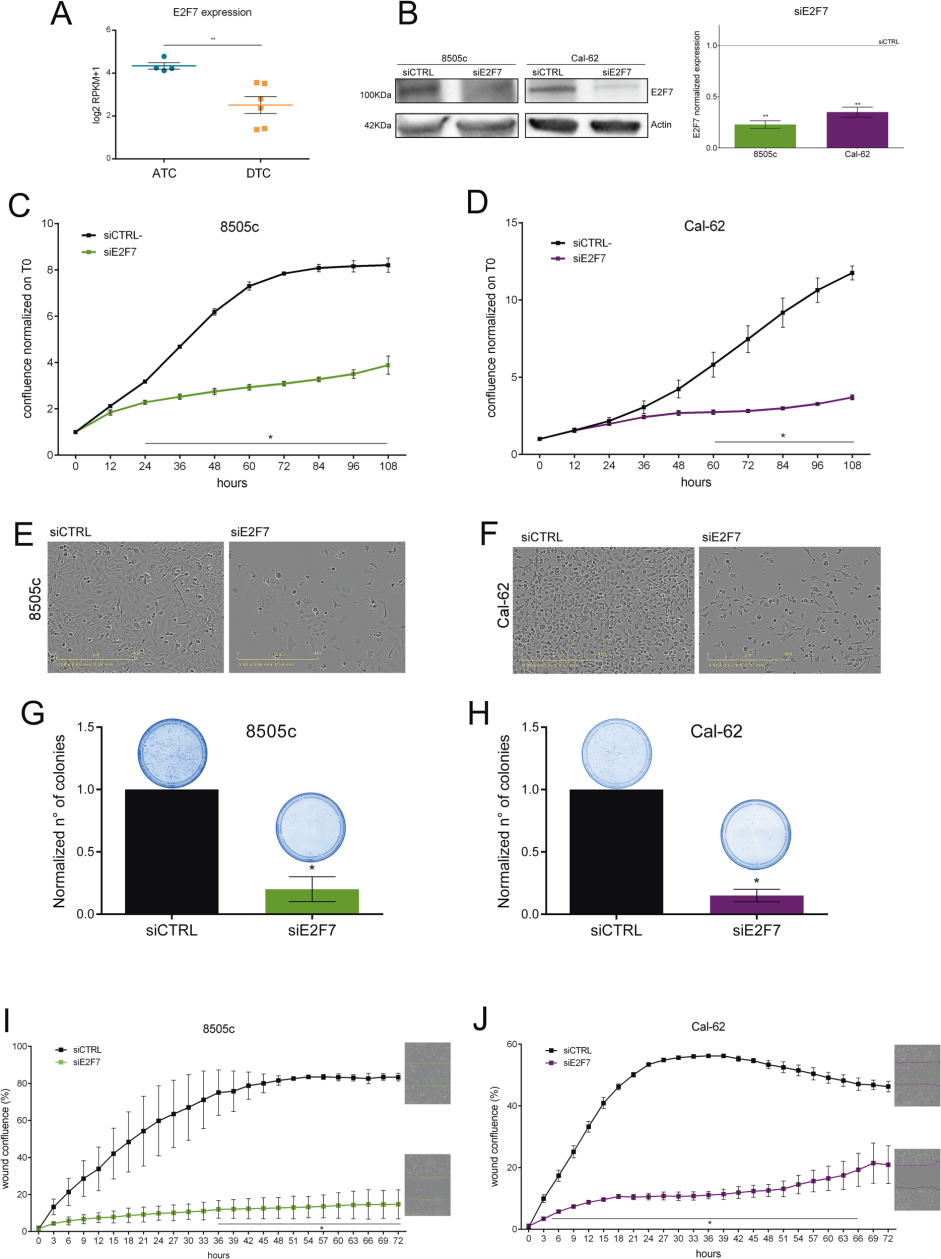
E2F7 is required for cell division progression in ATC. **A**, **B** Assessment of E2F7 expression in different ATC and DTC cell lines from the CellMiner database, expressed as log2RPKM **A** and in 8505c and Cal-62 cells 48 h after E2F7 silencing by qRT-PCR and Western blot **B**. Effects of E2F7 KD on proliferation **C**, **D**, cell morphology **E**, **F**, colony formation **G**, **H**, and migration **I**, **J** of 8505c and Cal-62 cells. Light microscopy representative images show cells at experimental endpoint (108 h for proliferation and 72 h for migration) with scale bar of 400 µm. Graphics represent means and SD of two independent biological replicates *p < 0.05, **p < 0.005.

E2F7 was the only TF emerging from this analysis. To consolidate these data, we evaluated E2F7 expression in thyroid cancer-derived cell lines, using the CellMinerCDB database. In accordance with our data, ATC-derived cell lines showed higher E2F7 expression compared to DTC-derived cells (Fig. 3A). To functionally assess E2F7 role in ATC, we knocked-down (KD) its expression in two ATC-derived cell lines, Cal-62 and 8505c, using two independent siRNAs (Fig. 3B, Supplementary Fig. 1D, Supplementary material). Noticeably, E2F7 KD led to a complete block of proliferation (Fig. 3C, D, Supplementary Fig. 1E), deeply affected cells’ morphology (Fig. 3E, F) and dramatically abolished clonogenicity in both cell lines (Fig. 3G, H). E2F7 KD also strongly reduced migration ability of both ATC cell lines (Fig. 3I, J, Supplementary Fig. 1F), further supporting a crucial role of this TF in sustaining the oncogenic program driving ATC evolution.

### E2F7-related gene program in ATC is linked to cell cycle progression and DNA repair

**Fig. 4:**
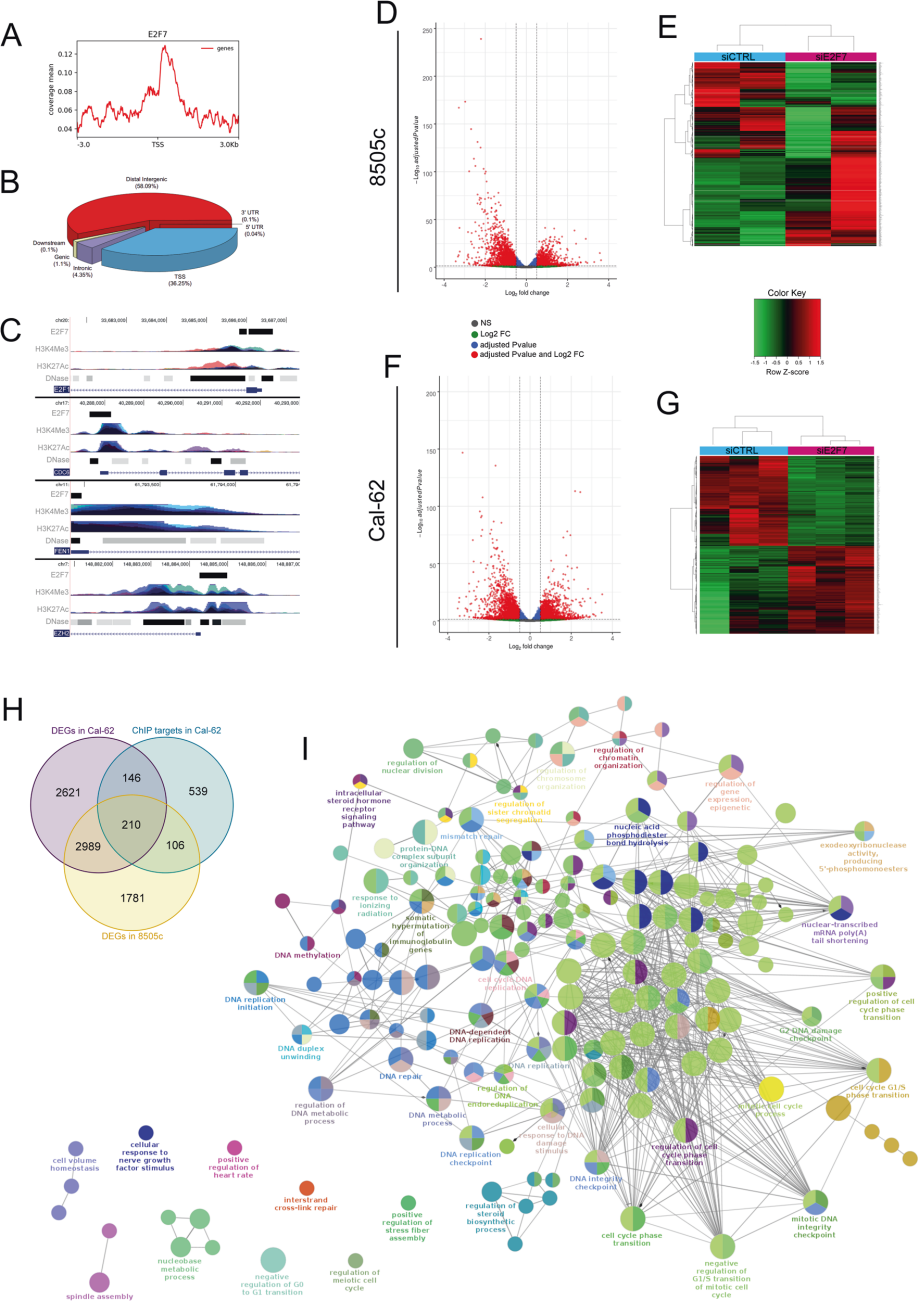
E2F7 target genes encompass a specific network of pathways. **A** Distribution of E2F7 ChIPseq peaks around TSS in Cal-62 cells, **B** Genome Browser visualization of E2F7 ChIP-seq peaks on the promoter of CDC6, E2F1, EZH2 and FEN1 target genes in Cal-62 cells. H3K4Me3, H3K27Ac and DNase tracks are also shown, to highlight promoter regulatory regions. **C** Genomic distribution of E2F7 peaks in Cal-62 cells. **D**–**F** Volcano plot displaying significantly DEGs (adjusted p-value and log2 FC) between cells transfected with siE2F7 and siCTRL, for 8505c and Cal-62 respectively. **E**–**G** Heatmap depicting hierarchical clustering based on DEGs whose read counts are Z-score normalized. Unsupervised hierarchical clustering was performed between cells transfected with siE2F7 and siCTRL, for 8505c and Cal-62 respectively. Color intensity for each gene shows Z-score values ranging from red for up-regulation and green for down-regulation. **H** Venn diagram showing the intersection between DEGs from RNA-seq of 8505c and Cal-62 cells and E2F7 direct target genes obtained by ChIP-seq on Cal-62 cells. **I** GO_BP network of the 210 genes defining the core of E2F7-mediated transcriptional program common to 8505c and Cal-62, modeled with Cytoscape app ClueGO.

To better understand the transcriptional network directly regulated by E2F7 in ATC cells, we performed a Chromatin ImmunoPrecipitation assay followed by deep-sequencing (ChIP-Seq) on Cal-62, to map the genome-wide distribution of its DNA binding (Fig. 4A), thus identifying 2458 significantly E2F7- enriched regions. Peaks distribution indicates that E2F7 preferentially binds to distal intergenic regions (58.09%) and Transcription Starting Sites (TSS) (36.25%), while its binding on intronic (4.35%), genic (1.1%), downstream, 3′ and 5′ UTRs (≤0.1%) was almost negligible (Fig. 4B). Peak to target assignment identified 1001 E2F7 putative target genes. Many known E2F7 target genes were present, such as *CDC6*, *E2F1, EZH2* and *FEN1*, supporting the specificity of our analysis [18, 24] (Fig. 4C). RNA-sequencing analysis on E2F7 KD and control (CTRL) cells was conducted in both cell models (Cal-62 and 8505c) to sort among the list of putative targets, those significantly affected by this TF. A total of 5086 differentially expressed genes (DEGs) were detected by this analysis in 8505c. Of these, 2651 (52%) were downregulated and 2435 (48%) upregulated (Fig. 4D, E). Similarly, 5966 DEGs were detected in Cal-62, of which 3014 (51%) downregulated and 2952 (49%) upregulated (Fig. 4F, G). 210 genes resulted by merging transcriptional data (from both cell lines) with the ones from the ChIP-seq analysis, defines the core of E2F7 transcriptional program in ATC (Fig. 4H, Supplementary Table S10).

Enrichment analysis performed on GO, KEGG and Reactome databases revealed that these genes were enriched in 4 functional macro-categories: Cell Cycle, DNA replication, DNA transcription, and DNA damage/repair and are strictly correlated (Fig. 4I).

**Fig. 5:**
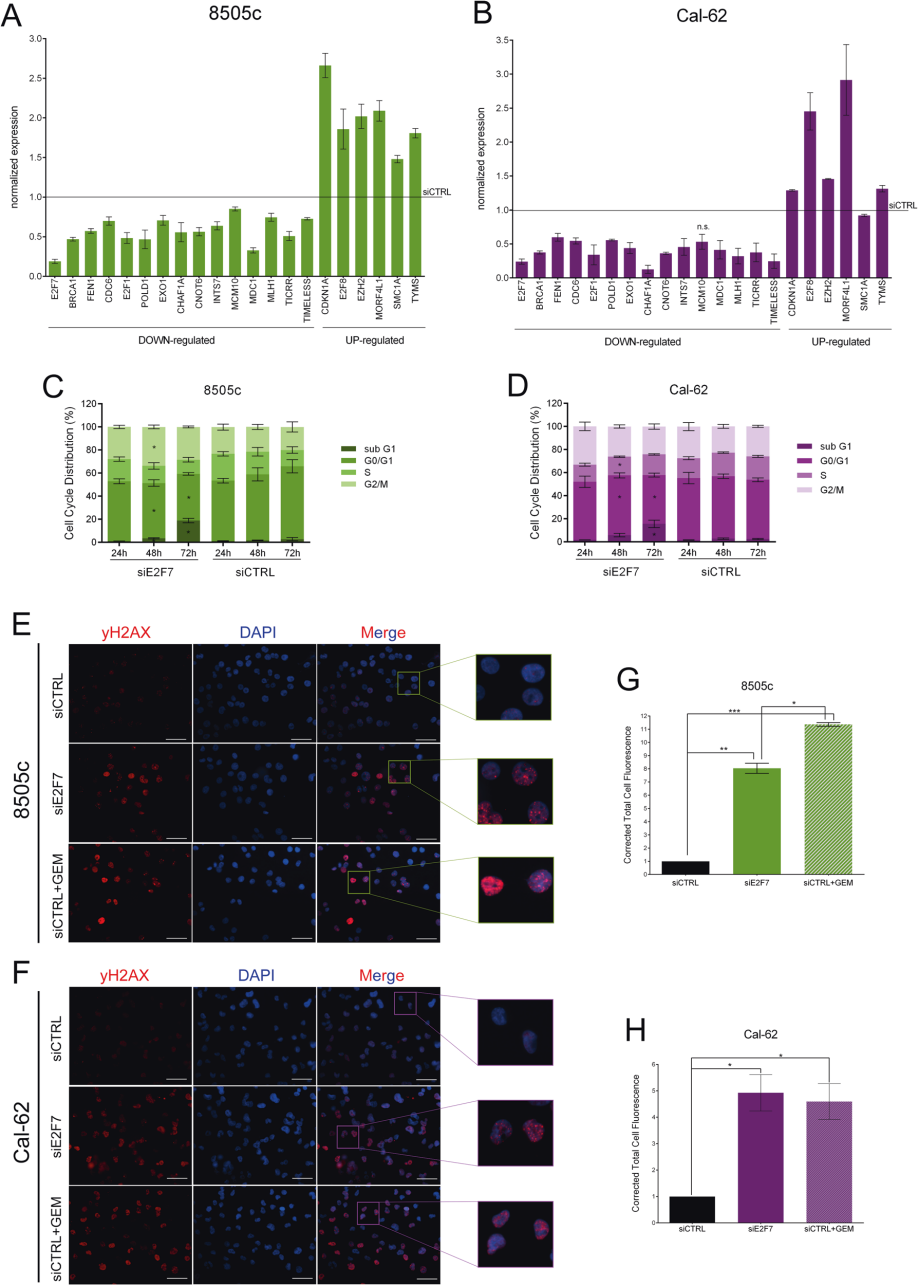
Validation of E2F7 role on cell cycle and DNA repair. **A**, **B** Validation of the 20 top-scoring DEGs from the intersection of RNA-seq and ChIP-seq experiments by qRT-PCR on 8505c and Cal-62 cells 48 h after siRNA transfection, normalized on siCTRL. All the values are statistically significant (adjusted *p*-value < 0.05) except where “n.s.” is indicated. **C**, **D** Cytofluorimetric analysis of 8505c and Cal-62 cells after E2F7 silencing for the evaluation of cell cycle phases distribution. **E**, **F** Immunofluorescence staining of γH2AX (red) in 8505c and Cal-62 48 h after E2F7 silencing. Gemcitabine was used as positive control for γH2AX staining. Nuclei are stained with DAPI (blue). Scale bar 50 µm. **G**, **H** Corrected Total Cell Fluorescence of 8505c and Cal-62 siE2F7 or siCT + gemcitabine cells stained for γH2AX, normalized on siCTRL cells, performed with ImageJ. Graphics represent means and SD of two or four independent biological replicates, *p < 0,05, **p < 0.005.

A panel of 20 genes representative of these four categories, were validated in an independent set of experiments, in both 8505c and Cal-62 cells (Fig. 5A, B).

To functionally confirm these data, we investigated the effect of E2F7 KD on two of the major biological functions identified by our analysis. First, we analyzed the cell cycle progression, and found an accumulation of cells in G1 phase with subsequent increase of the subG1 fraction, compared to control cells (Fig. 5C, D). Then, we evaluated the role of E2F7 on DNA repair. After siRNA transfection, both 8505c and Cal-62 underwent immunofluorescence for the S139 phosphorylated form of H2AX (γH2Ax), marker of DNA damage. Gemcitabine treatment was used as positive control. γH2Ax signal was significantly detectable in cells silenced for E2F7 at the same extent of control cells treated with gemcitabine, while in control cells was barely detectable (Fig. 5E–H).

Together, these results indicate that E2F7 plays a crucial role in the regulation of cell cycle and DNA damage and repair, in ATC cells. We defined 210 genes as the minimum core of E2F7-mediated transcription program guiding the progression of thyroid cancer cells towards a more aggressive phenotype. This contrasts with the onco-suppressive role of E2F7 described in other settings, opening the possibility to reconsider its activity in anaplastic cancers.

## Discussion

Anaplastic thyroid cancer accounts for 40% of thyroid cancer deaths and remains with no effective therapeutic options [25]. The relationship between DTC and ATC has long been debated [6, 26]. The hypothesis that ATCs originate from the de-differentiation of pre-existing DTCs made its way, but the molecular mechanisms that trigger this process and sustain ATCs aggressiveness remain unclear [7, 27]. Characterization of molecular drivers of ATC aggressiveness is mandatory also in the attempt to identify druggable targets, but these studies are restrained by samples rarity. In the present work, we performed a meta-analysis combining the highest reported number of publicly available gene expression datasets comprising 279 samples profiles, including 50 ATCs, to perform a robust analysis of the gene program underlying ATC onset, and validated it in a cohort of 23 FFPE samples, from our Institutional archive, using an independent technology.

Our data provide consistent evidence supporting the evolution of ATC from DTC through a complex gene expression reprogramming, leading to the progressive acquisition of features required for uncontrolled proliferation and metastatic spreading. We identified three signatures of DEGs clearly describing the de-differentiation processes leading normal thyroid cells to become DTC and DTC to ATC. Interestingly, the signature defining DTC-ATC transition is strongly related to cell cycle progression and proliferation thus perfectly fitting the aggressiveness features describing ATCs. Among the 210 DEGs, we found many genes known to be related to ATC progression, while others that had not previously been related to ATC. Our analysis suggests that the upregulation of a group of genes like the ones of the ATC-associated gene module, may serve to ATCs as an effective strategy to acquire proficient functional alterations [5]. We found E2F7 as the only TF in the gene module associated to ATC, thus identifying it as a potential candidate as driver of this transcriptional program. E2F7 is an atypical member of the E2F family. In its canonical function, it is highly expressed during mid to late S-phase and, acting as a repressor, it orchestrates negative feedback turning off the E2F-driven G1/S target genes and restraining cell cycle progression [15, 18]. Besides, E2F7 is involved in DNA damage repair and genomic stability regulation. It transcriptionally controls many DNA-repair relevant genes and its KD, alone or in combination with E2F8 KD, has been linked to increased cytotoxicity of DNA-damaging agents [19, 20]. For its role in promoting sensitivity to anti-cancer drugs and in inhibiting angiogenesis E2F7 is primarily acknowledged as tumor suppressor [14, 28, 29].

The balance between the expression of E2F7 and other E2F factors is well described also in differentiation processes during both development and cancer progression and aggressiveness. E2F7 in different settings promotes or restrains differentiation mechanisms, underlining its ability of playing multiple and often discordant functions depending on the cellular context in which it is operating [30–34]. This characteristic is common to many other cancer-related TFs that often have key roles during development and exploit similar mechanisms during cancer progression. Indeed, Weijts et al. recently described an unexpected E2F7 function in promoting blood vessels formation in zebrafish embryos. Forming a transcriptional complex with E2F8 and hypoxia inducible factor 1 (HIF1) it binds and transcriptionally activates the promoter of the vascular endothelial growth factor A (VEGFA), key factor guiding angiogenesis [35]. Moreover, Zhou and colleagues found a correlation between high levels of E2F7 and poor disease-free survival in thyroid cancer patients and demonstrated that E2F7 silencing restrain proliferation of PTC cell lines [36]. Our data are in line with this “non canonical” function of E2F7 in supporting cancer progression. Indeed, we showed that E2F7 partake to ATC evolution by ensuring cell cycle progression and protection of cells from DNA damage and cell death. Loss of E2F7 expression induces a dramatic reduction of cell proliferation, cells remain stuck in G1 phase and then undergo apoptosis. Moreover, typical aggressiveness features such as migration and ability to form colonies from single cell are impaired upon E2F7 silencing. Our omics profiling showed that as in other contexts, in ATC cells E2F7 works both as repressor or activator of gene expression. Coherently with the phenotypic changes observed, ChIP-Seq and gene expression analysis of E2F7 KD ATC cells demonstrated that E2F7 controls genes involved in cell cycle progression and DNA replication, transcription and repair confirming that its pro-tumoral function in this context is associated with its transcriptional activity. Differently from previous observation, in the context of ATC, we did not observe redundancy and/or compensation between E2F7 and E2F8 [14]. Even if E2F7 KD led to a significant increase in E2F8 expression (Fig. 5A, B), this cannot rescue the phenotype imposed by loss of E2F7, supporting a non-overlapping function of these factors in this setting.

These results suggested that the transcriptional program mastered by E2F7 is fundamental for the de-differentiation process guiding the rise of ATC from indolent lesion. Genes identified as E2F7 target in this context, are mostly well-known players of cell cycle progression and genome stability keepers. This suggest an oncogenic role for E2F7 which may be able to orchestrate the maintenance of DNA integrity and high levels of duplication and transcription thus sustaining high proliferation and adaptability needed for anaplastic transformation.

In conclusion, this study provides new insights into the biology of highly aggressive and therapy orphan thyroid cancer, by integrating information from many gene expression datasets, merging this information into a gene-network model, and validating this model in human samples. We identified a robust signature describing the molecular processes underneath DTC de-differentiation and we identified E2F7 as a master regulator of this transcriptional program. We are aware of the preliminary nature of our observations and further experiments are needed to fully understand the molecular mechanisms under E2F7 regulation in the context of ATC. However, the validity of our data strongly relies on the in vivo observation conducted on two different cancer patients’ cohorts, adding a new piece in the complex puzzle of ATC onset.

## Materials and methods

### Data and processing

Gene expression arrays of the Human Genome U133 Plus 2.0 (Affymetrix) platform were retrieved from The National Center for Biotechnology Information GEO database (Supplementary Table S1). The dataset contained ATC (*n* = 50), PTC (*n* = 102) and normal thyroid (*n* = 127) tissue samples [4, 37–43]. We performed background correction, quantile normalization and expression calculation using the Robust Multichip Average method implemented in the R/Bioconductor Affy package. After data normalization, samples were visualized by Principal Component Analysis based on the expression levels of all probes, to check for overall database homogeneity.

### Differential expression analysis

For each comparison (ATC vs DTC, ATC vs normal, and DTC vs normal), DEGs were detected using Student’s T-test followed by false discovery rate (FDR) multi-test correction with the Benjamini-Hochberg’s method.

Genes with at least one probe significantly deregulated (adjusted *p*-value < 0.05) in all comparisons were considered as deregulated (DEGs). From the list of DEGs we defined two groups: Trend Up and Trend Down, which were composed by genes that presented an increasing (Trend Up) or decreasing (Trend Down) trend in their expression levels (normal-DTC-ATC). Genes presenting more than one probe significantly deregulated but with discordant expression trend were excluded from the groups.

From the list of DEGs, we defined two groups: Trend Up and Trend Down, which were composed by genes resulted as DEGs in all comparisons and that presented an increasing (Trend Up) or decreasing (Trend Down) trend in their expression levels. The Trend Up and Trend Down lists presented 2571 and 3108 genes, respectively. Assuming the hypothesis of two steps of cancer progression, we derived gene signatures that represented: the most deregulated genes in the first step, i.e., normal to DTC transition (Up S1 and Down S1). The most deregulated in the second step, i.e., DTC to ATC transition (Up S2 and Down S2); and those deregulated through the entirely process of cancer progression (Up S3 and Down S3). The definition of these signatures was based on the standardized absolute fold change (sFC) values (calculated as (FC-average FC)/standard deviation) in the comparisons DTC vs. normal and DTC vs. ATC. For the “Up S1” signature: sFC (DTC vs. normal) > 4 and sFC (ATC vs. DTC) < 2; for the “Up S2” signature: sFC (DTC vs. normal) < 2 and sFC (ATC vs. DTC) > 4; “Up S3” signature: sFC (DTC vs. normal) > 2 and sFC (ATC vs. DTC) > 2; “Down S1” signature: sFC (DTC vs. normal) < −4 and sFC (ATC vs. DTC) > −2; “Down S2” signature: sFC (DTC vs. normal) > −2 and sFC (ATCvs. DTC) < −4; “Down S3” signature: sFC (DTC vs. normal) < −2 and sFC (ATC vs. DTC) < −2.

DEGs included in S1, S2, S3 were characterized by gene ontology (GO) biological processes using the R/Bioconductor packages topGO.

### Network analysis and gene module identification

Level of interaction between signature genes were obtained by mapping them into the STRING functional interaction network database (string-db.org), defining as gene modules the largest connected components formed by the gene signatures.

### Patients selection and RNA extraction

The archive of the Pathology Unit of our Institution includes more than 3800 primary thyroid cancer samples, diagnosed between 1975 and 2021 and staged according to the AJCC Cancer Staging Manual (7th edition). Of these, 38 were classified as ATC. For 23 of these samples, surgical formalin-fixed and paraffin embedded (FFPE) material was available for the gene expression. A total of 14 of these lesions also showed a differentiated component, while 9 were pure ATCs. For mixed samples, ATC and DTC components were microdissected and treated as separate samples. Supplementary Table S2 shows samples used for Nanostring analysis.

### Gene expression analysis by nanostring technology

Nanostring custom panel was designed including 17 and 21 genes from the Trend Up signatures S2 and S3, respectively. Nanostring experimental procedures and data analysis were conducted by Synlab Italia, Laboratory of Genetic and Molecular Biology, Pordenone, Italy. A total of 300 ng of total RNA for each sample were used for the analysis. Normalized gene expression values were obtained subtracting background and correcting for a coefficient calculated on positive controls and housekeeping genes.

### Cell cultures

Cal-62 and 8505c, a kind gift of Dr. Massimo Santoro (University of Naples, Italy), were cultured respectively in DMEM and in RPMI growth medium added with 10% fetal bovine serum (Thermo Fisher Scientific, Waltham, MA, USA) and 1% penicillin – streptomycin (Euroclone, Milan, Italy), at 37 °C/5% CO_2_. All cell lines were routinely tested for Mycoplasma contamination using Lonza Mycoalert Mycoplasma Detection Kit (Euroclone, Milan, Italy). Authentication by SNP profiling at Multiplexion GmbH (Heidelberg, Germany) was performed in January 2019 for Cal-62 and August 2020 for 8505c.

### siRNA transfection

For silencing experiment, 8505c and Cal-62 cells were reverse transfected with RNAiMax Lipofectamine (Thermo Scientific, Waltham, MA, USA) 24 h prior to seeding for subsequent experiments. RNA for RNA-seq experiments and their validation were collected 48 h after transfection. siRNAs used were Silencer siRNAs specific for E2F7 (ID#1: HSS135118 and ID#2:149428) and control Silencer Select RNAi Negative Control (Thermo Scientific, Waltham, MA, USA) at a final concentration of 15 nM.

### RNA extraction and quantitative real time PCR

Total RNA was extracted with Maxwell®RSC simplyRNA Cells (Promega, Madison, WI, USA) and retrotranscribed with iScript cDNA kit (Bio-Rad, Hercules, California, USA). Quantitative Real-Time PCR (qRT-PCR) was performed using Sso Fast EvaGreen Super Mix (Bio-Rad, Hercules, California, USA) in a CFX96 Real Time PCR Detection System (Bio-Rad, Hercules, CA, USA). Relative expression of target genes was calculated using the ΔΔCt method by normalizing to the reference gene expression (Actin). See Supplementary Table S3 for qRT-PCR primers.

For FFPE patients’ samples, total RNA was extracted using Maxwell RSC RNA FFPE KIT (Promega, Madison, WI, USA) and quantified using QUBIT RNA HS ASSAY KIT (Thermo Fisher Scientific, Waltham, MA, USA).

### Proliferation and wound healing assay

For proliferation assays, cells were seeded in 96-well plates (5000 cells/well in 4 wells for condition), and their confluence was evaluated every 12 h. For wound healing experiments, cells were seeded in IncuCyte® ImageLock 96-well plates (24000 cells/well in 5 wells for condition) (Sartorius AG, Goettingen, Germany), scratch wounds were created using the IncuCyte® WoundMaker (Sartorius AG, Goettingen, Germany), following manufacturer’s instructions. Migration was assessed through the evaluation of wound density every 3 h. Prior, cells were treated for 2 h with Mitomycin-c 10 µg/ml (Merck KGaA, Darmstadt, Germany) to impair proliferation.

Analysis was performed with the Incucyte® Live-Cell Analysis Systems (Model S3; Sartorius AG, Goettingen, Germany). Data were acquired using ×10 and ×20 objective lens in phase contrast. Settings used for each type of analysis are reported in Supplementary Table S4.

### 2D colony assay and cell cycle analysis

For colony formation, 5000 cells were plated in a 10 cm petri dish and allowed to form colonies for 7 days. Then, colonies were fixed with methanol, colored with Crystal Violet (Merck KGaA, Darmstadt, Germany) and counted with ImageJ software.

For cell cycle analysis, the hypotonic propidium iodide (PI) method [44] was used. Flow cytometry analysis were performed with FACS Canto™ II Cell Analyzer (BD Biosciences, Franklin Lakes, NJ, USA).

### Western blot and immunofluorescence

For western blot experiments, cells were lysed with PLB (Promega, Madison, WI, USA) supplemented with Protease Inhibitors cocktail (Bimake, Houston, TX, USA). 30 µg of total lysate were analyzed by SDS–PAGE using Bio-Rad apparatus (Bio-Rad, Hercules, CA, USA). Immunoblot detection was performed with specific primary antibodies 1 h to overnight incubation followed by 1 h incubation with the appropriate HRP-conjugated secondary antibodies (GE Healthcare, Piscataway, NJ, USA) and Clarity Western ECL substrate (Bio-Rad, Hercules, CA, USA). Full and uncropped western blots are presented in Supplementary Material. For immunofluorescence staining, cells were seeded in 4 well Cell Imaging slides (Eppendorf, Hamburg, Germany) then fixed in 4% PFA in PBS for 15 min at room temperature, permeabilized with 0.1% Triton in PBS 2% BSA for 2 min and blocked with 20% FBS in PBS 2% BSA for 1 hour. Next, cells were stained with primary antibody for 2 h at room temperature then with appropriate secondary antibody conjugated with AlexaFluor 555 (Thermo Fisher Scientific, Waltham, MA, USA). See Supplementary Table S5 for antibodies used. Images were captured with Nikon Eclipse microscope (Nikon, Chiyoda, Japan) using ×20 and ×40 magnification and are representative of at least two experiments.

Gemcitabine (Merck KGaA, Darmstadt, Germany) was added 48 h after siRNA transfection for 4 h at a final concentration of 200 nM to obtain a positive control for γH2AX staining.

### RNA-sequencing

Sequencing quality was assessed using the FastQC v0.11.8 software (www.bioinformatics.babraham.ac.uk/projects/fastqc/), showing on average a Phred score per base >34 in each sample. Raw sequences were aligned to the human reference transcriptome (GRCh38, Gencode release 35) using STAR version 2.7 and gene abundances were estimated with RSEM algorithm (v.1.3.1). Differential expression analysis was performed using DESeq2 R package, considering a False Discovery Rate (FDR) of 5% and excluding genes with low read counts. Heatmap representation and unsupervised hierarchical clustering with a complete linkage method were exploited to graphically depict differentially expressed genes (FDR < 0.05). Significant deregulated genes underwent to enrichment analysis, performed on Gene Ontology Biological Processes, KEGG and Reactome pathways databases by ClueGO via Cytoscape (v.3.7.1) for a network representation of enriched pathways, using a significance threshold of 0.05 on *p*-value corrected for multiple testing using Benjamini–Hochberg’s method and a medium level of GO term network connectivity (kappa score ≥ 0.5).

### Chromatin immunoprecipitation (ChIP) and ChIP-sequencing

Cal-62 cells were crosslinked for 15 min with 1% formaldehyde (Merck KGaA, Darmstadt, Germany), lysed and sonicated using Bioruptor Pico Sonicator (Diagenode, Denville, NJ, USA) to obtain 100–200 bp chromatin fragments as previously described [45]. Chromatin was precipitated using Dynabeads Protein G magnetic beads (Thermo Fisher Scientific, Waltham, MA, USA) and anti-E2F7 or Rabbit IgG antibodies (Supplementary Table S5). A fraction equal to 0.25% of total chromatin was used as input. Samples were quantified with Qubit (Thermo Fisher Scientific, Waltham, MA, USA), and the quality was evaluated by Bioanalyzer (Agilent Technologies, Santa Clara, CA, USA). Libraries for sequencing were obtained following the NEBNext Ultra II DNA Library Prep Kit for Illumina (New England BioLabs, Ipswich, MA, USA) using 3–10 ng ChIP DNA as starting material. Triplicates were sequenced on Illumina NextSeq500 high-output cartridge (single stranded, reads length 75 bp-1 × 75).

### Sequencing data processing

Sequencing quality was assessed using the FastQC v0.11.8 software and raw reads were trimmed to remove residual adapters using Trimmomatic (v.0.39) software. Trimmed reads were aligned to the human reference genome (GRCh38/hg38 assembly) by using Bowtie2 version 2.3.5.1 with default settings. Picard tool (http://broadinstitute.github.io/picard) and samtools 1.9 v (http://samtools.sourceforge.net/) were respectively exploited to remove duplicates and unmapped reads from filtered reads, to retain only uniquely aligned reads for downstream analyses. In order to find the regions of ChIP-seq enrichment over background that were consistently enriched across replicates, peak calling was performed on each sample using MACS2 (v.2.1.3.3) with default parameters and a qvalue threshold of 0.05. Significant peaks (qvalue < 0.05) were then merged and only high confidence peaks that were enriched in at least 2 of 3 replicates were retained for defining the genomic binding profile of the considered factor. Average profiles for ChIP-Seq enrichment around the TSS for all known genes were generated using DeepTools.

The ChIPseeker R package was exploited to verify the genomic distribution of the obtained biding profile and for assigning peaks to the nearest genes, according to GRCh38/hg38 annotation, using a TSS window of ±3 Kb. Annotated genes were checked for biological and functional enrichment using Gene Ontology Biological Processes, KEGG and Reactome pathways databases via EnrichR package.

### Statistical analysis

Statistical analysis was performed using GraphPad Prism Software (version 6.01 for Windows, GraphPad Software, San Diego, CA, USA). Statistical significance was determined using the Student’s *t*-test. Each experiment was replicated two to three times.

## Supplementary information

Supplementary Figure 1 legend

Supplementary material

Supplementary Table S1

Supplementary Table S2

Supplementary Table S3

Supplementary Table S4

Supplementary Table S5

Supplementary Table S6

Supplementary Table S7

Supplementary Table S8

Supplementary Table S9

Supplementary Table S10

Supplementary Figure

## Data Availability

The GEO datasets analyzed in this study are reported in Material and Methods section and in Supplementary Table S1. The RNA-seq generated during the current study are available in the Array Express repository, E-MTAB-9788. The ChIP-seq generated during the current study are available in the Array Express repository, E-MTAB-12277.
