# Supplementary material for: Adding pieces to the puzzle of differentiated-to-anaplastic thyroid cancer evolution: the oncogene E2F7
Source: Cell Death Dis. 2023 Feb 10;14(2):99. doi: 10.1038/s41419-023-05603-8 (PMC9918458; doi:10.1038/s41419-023-05603-8)
Supplement: Supplementary file 1 — Supplementary Figure 1 legend [file 41419_2023_5603_MOESM1_ESM.docx]

**Supplementary FIG. S1.**

A) Heatmap showing the normalized expression levels of the 150 probes with highest variance across the entire dataset. Columns represent samples and rows represent single probes. The columns are labeled according to the study of origin (GEO accession number) of each sample. The dendrogram shows the results of a Hierarchical Clustering applied to the list of samples. B) Venn diagram showing the number of probes resulting differentially expressed (adjusted p-value< 0.05) in more than one comparison. The numbers inside parenthesis show the total number of significant probes in each comparison. C) Principal Component Analysis applied to the gene expression datasets showing a bigger distance of the GSE3678 samples dataset from the other datasets along second principal component (y axis). D) qRT-PCR and Western blot of 8505c and Cal-62 cells 48h after E2F7 silencing by siRNA#2. E-F). Effects of E2F7 KD by siRNA#2 on proliferation (E) and migration (F) of 8505c and Cal-62 cells. Graphs represent means and SD of representative experiments (from two independent experiments) performed as technical replicates (n=4 or n=5, respectively). *p<0,05, **p<0,005.
