# Supplementary figures and images for: Adding pieces to the puzzle of differentiated-to-anaplastic thyroid cancer evolution: the oncogene E2F7
Source: Cell Death Dis. 2023 Feb 10;14(2):99. doi: 10.1038/s41419-023-05603-8 (PMC9918458; doi:10.1038/s41419-023-05603-8)

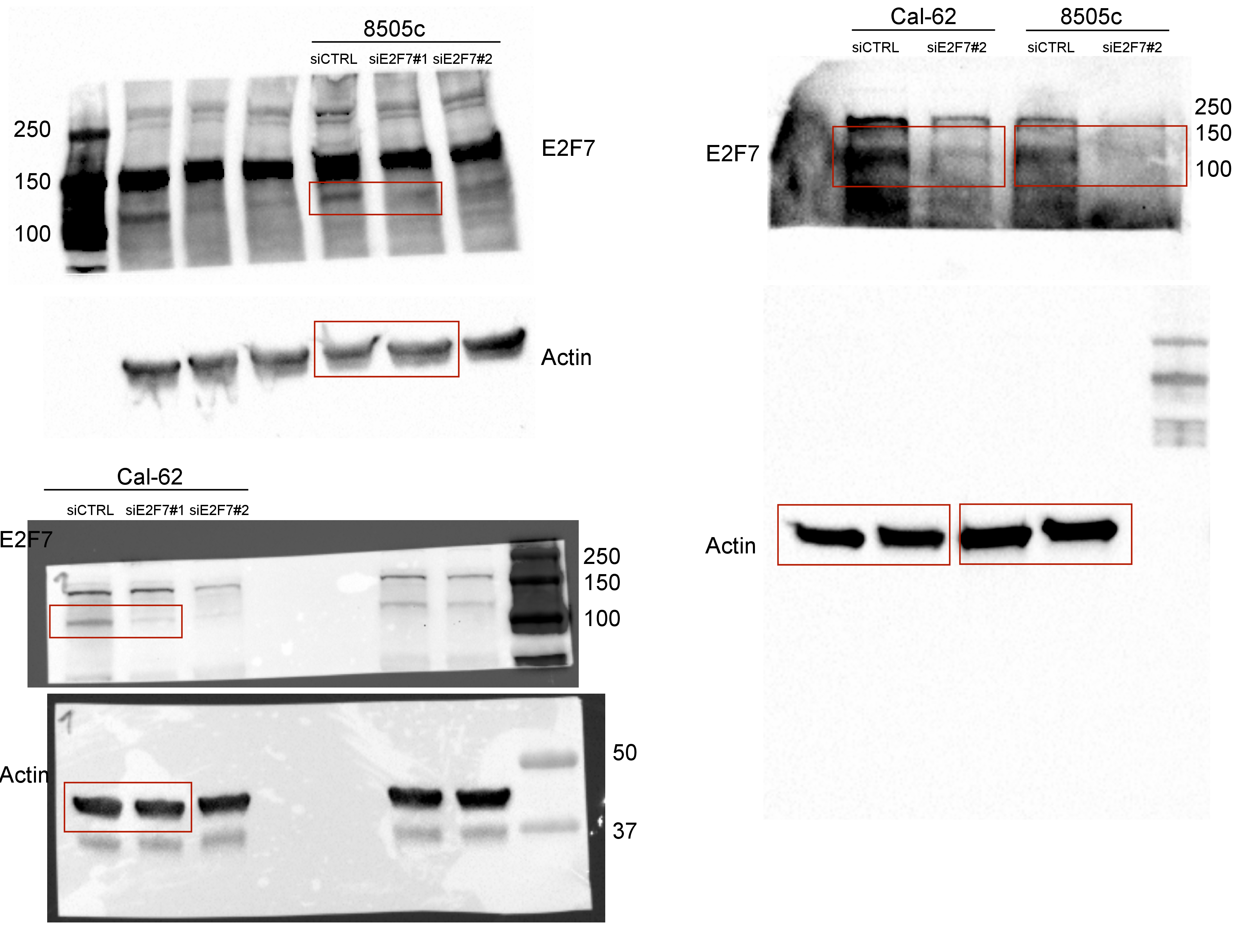

Supplement: Supplementary file 2 — Supplementary material [file 41419_2023_5603_MOESM2_ESM.tif]
